# Amino Acids Supplementation for the Milk and Milk Protein Production of Dairy Cows

**DOI:** 10.3390/ani11072118

**Published:** 2021-07-16

**Authors:** Jung-Eun Kim, Hong-Gu Lee

**Affiliations:** Department of Animal Science and Technology, Sanghuh College of Life Sciences, Konkuk University, Seoul 05029, Korea; sumzzzing@gmail.com

**Keywords:** protein metabolism, amino acids, milk protein, dairy cows

## Abstract

**Simple Summary:**

The composition of milk not only has nutritional implications, but is also directly related to the income of dairy producers. As regards milk’s composition, concerns around milk protein have emerged from the increased consumption of casein products. The synthesis of proteins in milk is a highly complex and high-cost process, because the conversion efficiency of dietary protein to milk protein is very low in dairy cows. Thus, some studies have increased milk protein by using protein supplements or a single amino acid (AA) supply. AAs are the building blocks of protein, and can also stimulate the protein synthetic pathway. This review mainly concerns the use of AAs for producing milk protein in high-producing dairy cows, particularly with methionine, lysine, and histidine. Understanding the mechanisms of AAs will help to promote milk protein synthesis in the dairy industry.

**Abstract:**

As the preference of consumers for casein products has increased, the protein content of milk from dairy cows is drawing more attention. Protein synthesis in the milk of dairy cows requires a proper supply of dietary protein. High protein supplementation may help to produce more milk protein, but residues in feces and urine cause environmental pollution and increase production costs. As such, previous studies have focused on protein supplements and amino acid (AA) supply. This review concerns AA nutrition for enhancing milk protein in dairy cows, and mainly focuses on three AAs: methionine, lysine, and histidine. AA supplementation for promoting protein synthesis is related to the mammalian target of rapamycin (mTOR) complex and its downstream pathways. Each AA has different stimulating effects on the mTOR translation initiation pathway, and thus manifests different milk protein yields. This review will expand our understanding of AA nutrition and the involved pathways in relation to the synthesis of milk protein in dairy cows.

## 1. Introduction

Milk’s composition relates to its nutritional value, but it is also directly linked to the income of dairy farms. Milk’s fat content has been scrutinized due to its nutritional value; however, as the preference of consumers has shifted towards protein products, studies on milk protein production have emerged.

Milk protein synthesis in dairy cows requires adequate supplies of energy and dietary crude protein (CP), specifically individual amino acids (AAs). Cows exhibit very low efficiency in converting nitrogen (N) into protein in its body or milk: the utilization efficiency of N is 25–35% [1]. According to National Research Council (NRC) (2001), the maximum yield of milk and milk protein is attributed to 22% of the dietary CP. However, due to the high cost of dietary protein sources, controlling single AAs may be a cost-effective strategy in high-producing dairy cows.

Although review papers on protein and amino acid metabolism in dairy cows have been introduced [2], this article focuses on AA nutrition for milk and milk protein production in dairy cows, with a specific focus on protein metabolism in the animal’s body, the importance of feeding with AAs, studies on AA supply, and the protein-synthetic pathway. As regards the AAs used, we mainly discuss methionine (Met), lysine (Lys), and histidine (His), which have been extensively studied previously.

## 2. Protein Metabolism in Ruminants

**Figure 1:**
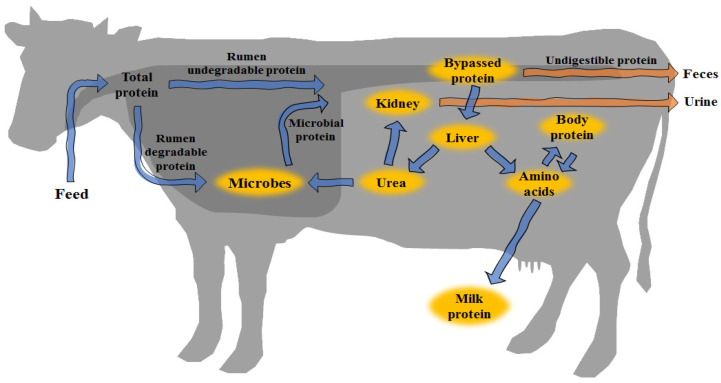
Protein metabolism of dairy cows.

Ruminants obtain nitrogen (N) sources that are available for metabolism from dietary intake, microbial proteins, and endogenous N (Figure 1). Dietary protein is divided into rumen-degradable protein (RDP) and rumen-undegradable protein (RUP). RDP is degraded by rumen microbes, then synthesized into microbial protein or partly bypassed, whereas RUP directly bypasses the rumen. The digesta, escaping the stomach and reaching the small intestine, is disassembled and absorbed into the blood stream, and undigested proteins are excreted as feces. The absorbed AAs pass through the liver to the kidneys, or flow into the blood stream. The AAs arriving at the mammary glands are synthesized into milk protein and subsequently secreted into milk.

## 3. Concept of AA Supplementation

The productive results in Section 3 are summarized in Supplementary Table S1.

Many studies have reported on oral administration and post-ruminal infusion as sources of protein for dairy cows. Soybean meal (SBM) and casein are typical protein supplements.

The effect of gradually increasing the level of SBM was studied [3,4]. The medium-protein group (formaldehyde-treated SBM inclusion; 15.4% of CP) showed a 1.4 kg/d increase in milk yield and a 48 g/d increase in milk protein yield compared to the low-protein group (no supplemented SBM; 11.3% of CP) when Holstein–Friesian cows received silage and three levels of protein concentration (ratio of 40:60) [4]. However, the high-protein group showed a similar milk yield to the low-protein group, and a similar milk protein yield to the medium-protein group. A linear increase in milk yield (26.6 to 28.0 kg/d) and milk protein yield (940 to 969 g/d) was observed when Swedish Red cows were fed grass silage-based diets (15.3% of CP), with various levels of SBM (17.3%, 19.0%, and 21.0% of CP) in their early lactation [3]. The supplementation of SBM to early-lactating Finnish Ayrshire, fed grass silage-based diets (17.0% of CP), significantly increased their milk yields and components by 3.0 kg/d, as compared to the control group [5]. In particular, the milk protein yield increased 113 g/d, and the milk protein concentration increased 0.14%, as compared to the control.

In a study on the abomasal infusion of sodium caseinate or enzymatically hydrolyzed casein into mid-lactating Holstein cows fed diets containing SBM (14.2% of CP) or SBM plus corn gluten meal (13.8% of CP), the sodium caseinate group showed increasing trends of milk yield (1.1 kg/d when SBM-fed and 1.2 kg/d when SBM plus corn gluten meal fed) and significant increases in milk protein yield (50 g/d), as compared to the saline-infused group [6]. However, the hydrolyzed casein group showed similar results to the saline group. The duodenal infusion of calcium caseinate produced more milk yield (1.01 kg/12 h), milk true protein concentration (0.90 g/kg), and milk true protein yield (36 g/12 h) in early-lactating Holstein cows as compared to those of the control group [7]. However, in Holstein cows fed basal diets containing 15.6% CP, the abomasal infusion of sodium caseinate did not affect lactational performance (numerical increases in milk yield and milk protein yield only) [8].

High-CP diets based on protein supplements may increase milk productivity. However, the problem is that N efficiency is also reduced [9]. Decreased N utilization efficiency causes huge losses to dairy farmers. Furthermore, N excreted in feces and urine (Figure 1) causes environmental pollution [10,11]. The price of protein supplements is relatively high when compared to other feed ingredients. In particular, the preference of SBM is high because it increases the productivity of cows, as described above. However, due to the high price of SBM, research on other protein feeds that can replace SBM has been reported. Some authors reported canola meal inclusion in dairy cow diets as a replacement for SBM [12,13]. When SBM or canola meal (CM) was included in diets, the CM group showed an increase [12] or no change [13] to milk and milk protein yield, as compared to those of the SBM group. In addition, studies on various other by-products or protein supplements that are used to replace SBM have been reported. Several protein products may be a good substitute for SBM. However, due to problems such as price and environmental pollution, the supplementation of excess protein sources should be limited. Therefore, it is necessary to carefully examine the supply of individual AAs, instead of surplus protein.

### 3.1. Balancing AA

When increasing milk protein, the level of a single AA supply is important, but it is also necessary to evaluate whether the supplied AAs are balanced. Concerns with balancing AAs have been increasing. Some authors have pointed out that increased milk production may be related to the increased or balanced AA supply that is a result of protein supplementation [3,5]. A decreased production of milk protein in dairy goats, due to deficiencies or imbalances of AAs, was observed in treatments employing abomasal AA mixture infusions with the deletion of Lys, arginine (Arg), Met, or His [14]. Similar results were reported when Holstein dairy cows received abomasal infusions of AA mixtures ( the ratio of milk protein) with Met, Lys, or His eliminated [15]. A deficiency or imbalance in His is induced by the dietary inclusion of feather meal (12.6%) [16]. Feather meal treatment, in the context of a standard protein diet, reduced both milk yield and milk protein yield by 7.4 kg/d and 331 g/d, respectively [16].

It is important to supplement deficient AAs for balancing the body’s AA pool well. Therefore, we must also assess which AAs are deficient, or risk producing an imbalanced environment.

### 3.2. Limiting AA

Essential AAs (EAAs) are synthesized by the animal itself at lower levels or not at all, and thus must be provided in the diet. For cows, the EAAs are the following: Arg, His, isoleucine (Ile), Leu, Lys, Met, phenylalanine (Phe), threonine (Thr), tryptophan (Trp), and valine (Val) [17]. Insufficient AAs, referred to as limiting AAs, are the AAs in the animal’s body pool that interrupt protein synthesis when they are deficient. Generally, Met and Lys are known as the co-limiting factors in corn and alfalfa silage-based diets [17,18], and His is known as a limiting AA when cows are fed grass silage-based diets [19,20,21,22]. Therefore, these three AAs have been widely reported on.

The first experiment on the intravenous infusion of Met, Lys, and His was reported in 1972 [23]. The infusion of 11.2 g/d Met increased milk protein yield by 30 g/d, as compared to controls, in early-lactating Holstein dairy cows fed corn silage-based diets. However, the infusion of His reduced the milk protein content, and the infusion of Lys did not affect milk protein. Met and Lys were shown to be co-limiting factors of milk protein synthesis when Holstein cows were fed corn-based diets (14.5% of CP) and receiving various combinations of 10 EAAs via abomasal infusion [17]. Another study suggested that Met and Lys were the two most limiting AAs when Holstein dairy cows in four different stages of lactation were duodenally infused with DL-Met, L-Lys, DL-Met plus L-Lys, or casein [18]. The infusion of both DL-Met and L-Lys increased milk protein content in early, mid, and late lactation, and milk protein yield in peak, early, and mid lactation, as compared to the DL-Met or L-Lys treatments. Met and Lys are the first-limiting AAs in corn and alfalfa silage-based diets in the United States because these AAs are laced in feed ingredients. Corn and SBM, typical feed ingredients, contain low levels of Lys and Met, respectively [1]. Hence, Met and Lys have been suggested as co-limiting AAs in United States diets.

Compared to those receiving the intravenous infusion of a mixture of four AAs (Met, Lys, His, and Trp), reduced milk yields (−3.2 kg/d) and milk protein yields (−159 g/d) were observed in the His-deprived group when feather meal, barley, and grass silage was offered to Friesian cows [19]. The continuous intravenous infusion to Frisian cows fed grass silage-based diets was conducted with a treatment of EAA (a composition of ten AAs in casein), three AAs (Met, Lys, and His), or His alone [20]. The results showed that all treatments increased the milk and milk protein yields when compared to the control, and no changes were observed between treatments. The abomasal infusion of His (6.5 g/d) increased milk yield (0.7 kg/d) and milk protein yield (26 g/d) in mid-lactating Finnish Ayrshire fed grass silage diets [22]. However, similar results were verified in infusion groups of His and Lys, His and Met, and three AAs, as compared to the His alone infusion group. Accordingly, His was considered a first-limiting AA when cows were fed grass silage diets. This is due to the low content of His in barley and feather meal, as well as in the rumen microbes [24].

Studies on other AAs, such as Ile, Leu, and Val, have also been performed. It is well known that branched-chain AAs (BCAAs; Ile, Leu and Val) are involved in protein metabolism [25]. The abomasal infusion of 150 g/d of BCAAs mixture did not affect milk or milk protein yields, as compared to the control group, when Holstein dairy cows were fed a total mixed ration (TMR) consisting mainly of corn and alfalfa hay (16.2% of CP) [26]. As regards diets based on cereal and grass silage, lactating Finnish Ayrshires showed no changes when infused abomasally with either a mixture of BCAAs and His or a mixture with one of BCAAs removed [27]. The abomasal infusion of His increased 0.8 kg/d of milk yield and 24 g/d of milk protein yield; however, no effect was observed when infusing Leu with His, as compared to the His-infused group, in early-lactating Finnish Ayrshire fed with grass silage [28]. The jugular infusion of Met and Lys or 2AAs plus BCAAs was reported when early-lactating Holstein cows were fed corn-based diets (16.1% of CP) [29]. Both groups presented increased milk protein content and increasing tendencies in milk protein yield; however, BCAA had no effect. An increase in milk yield (2.2 kg/d) and milk protein yield (40 g/d) was reported following the jugular infusion of Ile and Leu in Holstein cows fed corn-based diets (15.2% of CP) [30]. However, further experiments on the effects of BCAA on milk protein in dairy cows are still required.

### 3.3. Rumen-Protected AA

Artificially infused EAAs are a better choice for delivering AAs post-ruminally; however, this process is not applicable at the herd level. Thus, various rumen-protected (RP) AA products have been developed for supplying EAAs to the small intestine without being degraded by rumen microbes.

The RP-AAs, Met, Lys, or both, have been widely studied and used, with diverse results. Two trials with RP-Met were performed with Holstein dairy cows fed alfalfa and corn silage-based diets [31]. In trial 1, treatments of 17.3% of CP plus 5 g of RP-Met and 16.1% of CP plus 10 g of RP-Met significantly increased the milk yield (+1.9 kg), as compared to 18.6% of CP with no supplementation group; however, the milk protein yield was unchanged. The supplementation of diets containing 17.3% or 16.1% CP with 10 g of RP-Met resulted in similar milk yields and composition as compared to the no RP-Met group. In early-lactating dairy cows, fed 14.5% of CP, RP-Met (0.03% of diets) tended to decrease milk protein content due to the numerical decrease in milk protein yield [32]. RP-Lys, supplemented at approximately 94.4 g/d/cow, increased milk and milk protein yields (2.03 kg/d and 80 g/d, respectively) in early-lactating Holstein cows fed alfalfa- and corn-based diets (17.0% of CP) [33]. In the same study with mid-lactating cows, RP-Lys supply tended to increase milk and milk protein yields (0.82 kg/d and 20 g/d, respectively). However, similar milk production levels were also reported as being between 0 or 60 g/d of RP-Lys-supplemented groups, when early-lactating Holstein cows were fed corn silage-based diets (16.4% of CP) with 10 or 20% of dried distillers grains plus solubles [34].

The effects of RP-Met and RP-Met plus RP-Lys were tested in early-lactating Holstein dairy cows fed alfalfa hay and heated whole soybean-based diets (19.5% of CP) [35]. A linear increase was observed in both milk protein content and yield when 5.25 and 10.5 g/d of RP-Met were supplemented. However, the supplementation of RP-Met plus RP-Lys (11.5 and 14.7 g/d, respectively) did not present changes as compared to the 10.5 g/d RP-Met group [35]. Contrary to the experiment mentioned above, the supplementation of 40 g/d of RP-Met and Lys product increased both milk and milk protein yields, as compared to the 15 g/d RP-Met group, when Holstein dairy cows were fed corn-based diets containing 16.0% or 18.5% of dietary CP [36]. The tablet forms of RP-Met (18.2 g/d supply), RP-Lys (11.7 g/d), or both were supplied to early-lactating Holstein cows fed maize silage and cereal-based diets (14.5% of CP) [37]. RP-Met or RP-Lys alone caused no changes in milk and milk protein yields; however, RP-Met plus RP-Lys increased the milk protein yield.

For estimating the effects of RP-Met on milk production and milk protein, a meta-analysis was conducted on papers that used two widely studied products: Mepron (Evonik Industries, Hanau, Germany) and Smartamine M (Adisseo, Antony, France) [38]. The analysis predicted that RP-Met supply would lead to 0.07% more produce and 27 g of true milk protein content and yield (true milk protein: milk CP × 0.94). In the same paper, the authors also analyzed whether AA deficiency and forage sources would affect true milk protein, and the results suggested that both true milk protein content and yield would be increased, regardless of the state of the AAs (adequate or deficient Met, Met + Lys, or Met + Lys + one other AA) and the main forage source (alfalfa, corn silage, grass, or grass + corn silage). Overall, the aforementioned studies and meta-analysis indicate that supplementation with RP-Met has the potential to increase the milk protein yield of dairy cows in various conditions.

After considering His, as a potential AA, to be a first-limiting AA, the effects of RP-His with or without RP-Met and RP-Lys on milk production were investigated. Gradually increasing amounts of RP-His (0, 82, 164, and 246 g/d) were supplemented to mid-lactating Holstein dairy cows fed corn silage-based diets (15.1% of CP, containing 11 g/d of RP-Met) [39]. A tendency to increase was observed in both milk yield (linearly) and milk protein yield (quadratically), and an actual increase in milk protein yield (+50 g/d) was presented when 256 g/d of RP-His was supplied. Morris and Kononoff (2020) reported the effects of RP-Lys, RP-His, or both in Jersey dairy cows fed corn silage-based diets (17.1% of CP) with hydrolyzed feather meal (His-deficient source). Supplementation with 70 g/d of RP-Lys did not change milk production; however, a supply of RP-His (32 g/d) caused a significant increase in milk yield and a tendency towards an increase in milk protein yield [40]. In some studies, the effect of His was tested when metabolizable protein (MP)-deficient diets (MPDs) were offered to cows [41,42,43]. A supply of MPD (13.6% of CP; corn silage and alfalfa haylage-based diets) with RP-Met (30 g/d/cow) plus RP-Lys (100 g/d/cow) with or without RP-His (50 g/d/cow) increased milk protein yield as compared to the MPD with no supplementation group, and the resulting yield was similar, as compared to the MP-adequate (MPA; 15.7% of CP) diet group. However, compared to MPD with RP-Met and RP-Lys, the effect of RP-His was not verified [43]. The effects of MPD, slow-release urea, RP-Met (30 g/d), and RP-His (50 g/d) on the productivity of dairy cows were estimated in Holstein dairy cows fed corn silage-based diets [42]. Milk protein production was not affected by MPD (14.8% of CP), MPD plus urea (15.8% of CP), or RP-Met, but it was affected by RP-His. Moreover, the supplementation of MPD with RP-His plus urea and RP-Met increased the milk protein yield (90 g/d) as compared to the MPA group (16.7% of CP). The same author studied MPD, RP-Met, Lys, and His [41]. The milk protein yield showed only numerical changes in each of the Met, Lys, and His alone groups as compared to MPD (average 14.5% of CP); however, supplied with mixture of three AAs to MPD, milk protein yield was increased 100 g/d. Due to the diversity of the effects and the limitations of these studies, further research on the effects of RP-His is still required. Research on other AAs, besides the three mentioned, has also been carried out (RP-Leu [44], RP-Phe [45,46,47], RP-Trp [48], RP-AAs (mixture of Lys, Ile, Val, and His in RP form) [49], etc.). Further studies on RP-AAs in various feeding types and under various animal conditions are required.

## 4. Effects of AA Supplementation on Mammary Translational Expression

**Figure 2:**
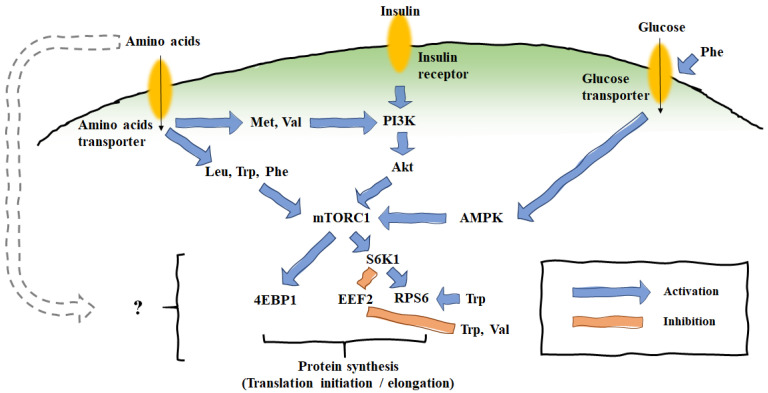
Mammalian target of rapamycin pathway.

For protein synthesis, the pivotal substances are as follows: AAs (building blocks), glucose (energy source), and insulin. Protein is synthesized by the mammalian target of rapamycin (mTOR) complex and its downstream pathway [50,51]. Due to the high-energy demands of protein synthesis, mTOR translational regulation is performed by the energy source, usually glucose [52,53]. When glucose is recognized by the AMP-activated protein kinase (AMPK), its activation inhibits mTOR. Insulin is essential to the cellular metabolism. Phosphoinositide 3-kinase (PI3K) is stimulated by insulin, and further stimulates protein kinase B (Akt). The AAs are not only building blocks of protein; they are also potential regulators of mTOR translational regulation [50,52,53]. In particular, leucine (Leu) is known to stimulate the mTOR pathway either indirectly or directly [54,55]. Once the mTOR complex is phosphorylated, the activity of the ribosomal protein S6 kinase 1 (S6K1) is blocked, and the activated ribosomal protein S6 (RPS6) enters for further translation processes. Meanwhile, mTOR phosphorylates the eukaryotic initiation factor (eIF) 4E binding protein 1 (4EBP1). Then, through the translation initiation and elongation stage, involving eIF and eukaryotic translation elongation factor 2 (eEF2), milk proteins are produced. An illustration of the basic mTOR pathway, supplemented with our recent in vitro results, is presented in Figure 2 [56,57,58].

The effects of AAs on the protein synthetic pathway have been assessed via the in vitro incubation of mammary cells and mammary biopsies taken from cows supplemented with AA. Due to the lack of experimental animals, many studies on milk protein synthesis with the mTOR pathway have been conducted in vitro, using immortalized or primary bovine mammary epithelial cells [56,57,58,59,60,61].

In our previous studies, AA treatments of immortalized bovine mammary epithelial cells (MAC-T cells) proceeded through different pathways [56,57,58]. The supplementation of 0.6 mM of L-Met in MAC-T cells was shown to increase β-casein expression and stimulate the PI3K pathway; however, an effect on mTOR was not observed [57]. eEFs were detected when 0.9 mM of L-Phe and 0.6 mM of L-Val were supplied to MAC-T cells; however, neither the mTOR nor the β-casein were altered in these treatments [58]. When 0.9 mM of L-Trp was added to the MAC-T cell medium, significant increases were observed in the mRNA expressions of mTOR, RPS6, and β-casein [56]. The inclusion of 0.15 mM of L-His increased β-casein expression, as compared to the control, when MAC-T cells were cultured in nutrient-restricted medium [60]. The effects of His, Lys, Met, and Leu on the mTOR pathway and β-casein were reported in Chinese Holstein mammary epithelial cells (CMEC-H cells) [59]. The expressions of mTOR, S6K1, RPS6, and β-casein increased, as compared to the control, following supplementation with 0.15 mM of His, 0.5 mM of Lys, 0.12 mM of Met, and 0.45 mM of Leu. In that study, His also increased the expression of 4EBP1, while Met decreased it [59]. The supplementation of Arg to primary mammary epithelial cells (pMEC) from Chinese Holstein cows increased the mRNA levels of four types of casein, mTOR, and S6K1, but decreased 4EBP1 [61].

The effects of EAAs and glucose infused by the jugular vein were reported when early-lactating Holstein cows experienced 22 h of nutrient deprivation [62]. Mammary biopsy samples taken after the treatment were analyzed for gene expression, and the results showed phosphorylated S6K1 (pS6K1) as a result of Leu, and phosphorylated RPS6 as a result of Leu and Met plus Lys. Although changes were observed in mTOR-pathway-related genes, the infusion of His, Leu, and Met plus Lys presented only a numerical increase in milk protein yield. Two studies were reported that investigated the effects of the abomasal infusion of EAAs (ratio of casein) on the milk protein yield and mRNA translation of mammary samples [63,64]. The elimination of His, Phe, BCAAs, Leu, and Lys from 10 EAAs significantly decreased the milk protein yield as compared to the 10-EAAs-infused group [63,64]. In one study, the phosphorylation of S6K1 (pS6K1/total S6K1) was enhanced following the removal of His as compared to supplementation with 10 EAAs [63]; however, no changes were observed in another study [64]. The jugular infusion of Arg with Lys, Met, Phe, and Ile (ratio of casein) increased milk yield (+2.71 kg/d), milk protein yield (+120 g/d), α-casein (+5.54 g/L) and κ-casein (+1.00 g/L) in Chinese Holstein dairy cows fed diets containing 14.08% of CP [65]. However, a significant increase in casein gene expression was only observed for αs_1_-casein and αs_2_-casein. In the case of mTOR and S6K1 expression following mammary biopsy, increases of more than 18-fold in mTOR and 5-fold in S6K1 were observed, as compared to the control; however, 4EBP1 expression was decreased.

Each of the AAs influence protein synthesis via different pathways and genes. Studies on mammary translational regulation via AAs in dairy cows are still required, as these will expand our understanding of milk protein production in dairy cows.

## 5. Summary and Conclusions

The supplementation of AAs to dairy cows is a possible method for increasing milk and milk protein yield. Supplying protein feed seems to be a more certain method by which to increase the cow’s productivity, as compared to other methods (see Table S1). However, high-protein feeds in dairy cow diets have several problems: high cost, low utilization due to the cow’s low efficiency, high N excretion, and environmental contamination such as eutrophication. Therefore, it is of great concern to control individual AAs, rather than supplying surplus protein only. The advantage of intravenous infusion is that we can incorporate AAs into the blood stream of the animal’s body, as we choose to target. Abomasal infusion can also supplement AAs according to the needs of the researcher; however, it is a relatively indirect method to affect productivity, when compared to intravenous infusion, because it passes the digestive tract. Both infusion methods are useful in experiments; nevertheless, they are not applicable in an industrial environment. The final way to supplement AAs to cows is to use RP-AAs. RP-AAs can mitigate insufficient single AAs in the body’s AA pool, but not excess protein. Therefore, it is believed that there is a possibility to maintain or increase the productivity of animals while lowering the CP content in their diets. However, as shown in Table S1, RP-AAs provide inconsistent results. Overall, although supplying RPAAs appears to be cost effective, further testing and development will be needed for actual applications with dairy cows. For dairy cows, the most-limiting amino acids are considered to be Met, Lys, and His. It mainly depends on the AA composition in their diets; thus, Met and Lys are the co-limiting factors in corn- and alfalfa-based diets and His is the first-limiting AA in grain and grass silage-based diets. Milk proteins are synthesized through the mTOR translation initiation pathway. Studies on milk protein stimulation using AAs, with analysis of mammary samples, have been conducted; however, remarkable results are still limited. Future research on AA nutrition and milk protein synthesis are required, and will provide more field-applicable understanding.

## Data Availability

No new data were created or analyzed in this study. Data sharing is not applicable to this article.

## Supplementary Materials

The following are available online at https://www.mdpi.com/article/10.3390/ani11072118/s1, Figure S1: Summarized productive results according to various feeding methods as described in Section 3.
