# Supplementary material for: Amino Acids Supplementation for the Milk and Milk Protein Production of Dairy Cows
Source: Animals (Basel). 2021 Jul 16;11(7):2118. doi: 10.3390/ani11072118 (PMC8300144; doi:10.3390/ani11072118)
Supplement: Supplementary file 1 [file animals-11-02118-s001.zip › animals-1295994-supplementary.pdf]

Supplemental Table S1. Summarized productive results according to various feeding methods as described in section 3.

Feeding methods			Outcome		
Methods	Treatment[1]	Dietary CP[2] (%)	Milk yield (kg/d)	Milk protein yield (g/d)	Reference
Diet	Soybean meal	15.3			[3]
	To meet 17.3% of dietary CP		+0.9	+19	
	To meet 19.0% of dietary CP		+1.4	+29	
	To meet 21.0% of dietary CP		+1.4	+26	
Diet	Soybean meal (to meet 15.4% of dietary CP)	11.3	+1.4	+48	[4]
Diet	Soybean meal (to be isonitrogenous)	17.1	+3.0	+113	[5]
Diet	Soybean meal vs. canola meal	14.7 or 16.6	+1.0 (canola meal)	+30 (canola meal) (milk true protein)	[12]
Diet	Feather meal (12.6% inclusion in diet)	19.2	-7.4	-331	[16]
Duodenal infusion	Calcium caseinate (743 g/d/cow)	14.1	+1.0 (kg/12 h)	+36 (g/12 h) (milk true protein[3])	[7]
Abomasal infusion	Sodium caseinate (To meet 15% of metabolizable protein)	15.6	-	-	[8]
Abomasal infusion	Amino acids mixture (ratio of milk protein)	9.3			[15]
	Deletion of Met		-	-	
	Deletion of Lys		-	-	
	Deletion of His		-	-	
Abomasal infusion	His (6.5 g/d/cow)	14.2	+0.7	+26	[22]
Abomasal infusion	BCAA (150 g/d/cow)	16.2	-	-	[26]
Abomasal infusion	His (6.5 g/d/cow)	14.6	+0.8	+24	[28]
Intravenous infusion	Met	No data			[23]
	11.2 g/d/cow		-	+30	
	24.8 g/d/cow		-	-	
	Lys				
	33.5 g/d/cow		-	-	
	65.9 g/d/cow		-	-	
	His				
	21.2 g/d/cow		-	-	

	50.3 g/d/cow		-	-	
Intravenous infusion	Mixture of Met, Lys, His, and Trp	No data			[19]
	Delition of His		-3.2	-159	
Intravenous infusion	Amino acids mixture (ratio of casein)	No data	+2.9	+107	[20]
	Met, Lys, His (7.1, 17.6, 6.0 g/d/cow)		+2.4	+108	
	His (6.0 g/d/cow)		+2.3	+60	
Intravenous infusion	Met, Lys	16.1	-	+130	[29]
	Met, Lys, BCAA		-	+129	
Intravenous infusion	Ile, Leu	15.2	+2.2	+40	[30]
RPAA[4]	RP-Met (0.03% of diets)	14.5	-	-	[32]
RPAA	RP-Lys (94.4 g/d/cow)	17.0	+2.03	+80	[33]
RPAA	RP-Lys (60 g/d/cow)	16.4	-	-	[34]
RPAA	RP-Met (5.25 g/d/cow)	19.5	-	+28	[35]
	RP-Met (10.5 g/d/cow)		-	+42	
	RP-Met, RP-Lys (11.5, 14.7 g/d/cow)		-	+59	
RPAA	RP-Met (40 g/d/cow)	16.0	-	-	[36]
		18.5	-	-	
RPAA (tablet form)	RP-Met (18.2 g/d/cow)	14.5	-	-	[37]
	RP-Lys (11.7 g/d/cow)		-	-	
	RP-Met, RP-Lys (18.2, 11.7 g/d/cow)		-	+64	
RPAA	RP-His	15.1 (containing 11 g/d/cow of RP-Met)			[39]
	82 g/d/cow		-	-	
	164 g/d/cow		-	-	
	246 g/d/cow		-	+50 (milk true protein)	
Diet	RP-Lys (70 g/d/cow)	17.1 (containing feather meal)	-	-	[40]
RPAA	RP-His (32 g/d/cow)		+0.9	-	
	RP-Lys, RP-His		-	-	
RPAA	RP-Met, RP-Lys (30, 100 g/d/cow)	13.6	-	-	[43]
	RP-Met, RP-Lys, RP-His (30, 100, 50 g/d/cow)		-	+100	
RPAA	Slow release urea	14.8	-	-	[42]
	+ RP-Met (30 g/d/cow)		-	-	

	+ RP-Met, RP-His (30, 50 g/d/cow)		-	+100	
RPAA	RP-Met (30 g/d/cow)	14.5	-	-	[41]
	RP-Lys (130 g/d/cow)		-	-	
	RP-His (120 g/d/cow)		-	-	
	RP-Met, RP-Lys, RP-His (30, 130, 120 g/d/cow)		-	+100	

[1]Arg = arginine; His = histidine; Ile = isoleucine; Leu = leucine; Lys = lysine; Met = methionine; Phe = phenylalanine; Thr = threonine; Trp = tryptophan; Val = valine; BCAA = branched-chain amino acids.

[2]Dietary CP = dietary crude protein.

[3]Milk true protein = milk protein × 0.94.

[3]RPAA = rumen-protected amino acids.
